# Optimization of canine interleukin-12 production using a baculovirus insect cell expression system

**DOI:** 10.1186/s13104-016-1843-7

**Published:** 2016-01-22

**Authors:** Cristiane Garboggini Melo de Pinheiro, Mayara de Oliveira Pedrosa, Naiara Carvalho Teixeira, Ana Paula Dinis Ano Bom, Monique M. van Oers, Geraldo Gileno de Sá Oliveira

**Affiliations:** Centro de Pesquisas Gonçalo Moniz, Fundação Oswaldo Cruz, Salvador, Bahia Brazil; Programa Nacional de Pós Doutorado-CAPES/Programa de Pós-graduação em Biotecnologia em Saúde e Medicina Investigativa, Centro de Pesquisas Gonçalo Moniz, Fundação Oswaldo Cruz, Salvador, Bahia Brazil; Laboratório de Macromoléculas, Bio-Manguinhos, Fundação Oswaldo Cruz, Rio de Janeiro, Brazil; Laboratory of Virology, Wageningen University, Wageningen, The Netherlands; Instituto Nacional de Ciência e Tecnologia de Doenças Tropicais (INCT-DT), Salvador, Brazil

**Keywords:** Interleukin-12, Dog, Protein expression optimization, Baculovirus

## Abstract

**Background:**

Interleukin-12 is an important cytokine in mediating cellular immune responses.

**Results:**

Recombinant single-chain canine IL-12 was produced in a baculovirus-insect cell system with the aim of conducting further studies on modulation of immune responses in dogs. To optimize the production of recombinant canine IL-12, a classical baculovirus and a modified vector (*chitinase A and v*-*cathepsin* knockout) were used containing a native or an optimized insert of canine IL-12. The optimized IL-12 construct contained the GP64 signal peptide and was synthesized with optimized codons for expression in *Trichoplusia ni* cells. Dot-blot and Western blot analysis showed the highest production levels of recombinant IL-12 protein by the use of the modified baculovirus vector containing the optimized insert, at a multiplicity of infection of five and at 48 h after infection. The recombinant cytokine was successfully purified and showed a good degree of purity, integrity, folding, and yield, with very little endotoxin contamination. Recombinant canine IL-12 induced IFN-γ in canine lymphocytes, indicating that it was biologically active.

**Conclusion:**

Therefore, this study describes an efficient method to produce adequate amounts of biologically active canine IL-12, useful for immunomodulation studies in dogs.

## Background

Interleukin-12 was originally described as natural killer cell stimulatory factor-NKSF for its ability to promote activation of NK cells [1]. This cytokine plays an important role in inducing IFN-γ production by T and natural killer cells [1, 2], and in differentiation of Th1 CD4 ± T cells [3, 4]. Studies have shown that IL-12 has a potential therapeutic benefit in cancer [5, 6], infectious and inflammatory diseases, and as a vaccine adjuvant [7–9]. Thus, production of recombinant IL-12 has raised great interest in the scientific community.

Interleukin-12 (IL-12) is a 70 kDa heterodimeric cytokine composed of two disulfide-bonded subunits—p40 and p35, [10–12]. The heterodimer (p70) is required for the biologic activity of IL-12 [13, 14]. In addition, only the posttranslational N glycosylated protein isoforms are biologically active [15]. Therefore, recombinant IL-12 should be produced in eukaryotic cells to display functional activity.

Okano et al. [16] cloned both subunits of canine IL-12 and described their expression in mammalian cells. Later, canine IL-12, showing biological activity, was cloned and expressed as a single chain protein (rsccaIL-12) in the green monkey cell line COS-7 cells. rsccaIL-12 was able to induce IFN-γ mRNA expression in peripheral blood mononuclear cells (PBMC) from healthy and visceral leishmaniasis sick dogs [17]. However, the production of tens of milligrams of recombinant proteins in mammalian cells, necessary for pre-clinical trials in dogs, would be costly and laborious.

A feasible alternative for producing adequate amounts of the recombinant protein for pre-clinical trials is the baculovirus-insect cell system, which is being used extensively to express heterologous genes in insect cells cultures or insect larvae [18]. This system allows for production of high levels of recombinant proteins with posttranslational modifications, including N-glycosylation. Recombinant IL-12 subunits, p35 and p40, of bovine [19] and equine [20] have been produced in insect cells, resulting in a bioactive IL-12 p70 heterodimer. Furthermore, Poot et al. [21] have produced canine IL-12 in baculovirus-insect cell system using classical recombinant baculovirus and adherent insect cell cultures. However, this report lacks detailed information regarding production and yield of canine IL-12.

This study was conducted to determine appropriate conditions to produce moderate amounts of canine IL-12 to assess in a near future the feasibility of inducing Th1 immune responses in dogs, which would be useful in developing vaccines and treating diseases caused by intracellular pathogens, allergic diseases, and cancers. Herein, to determine appropriate conditions to produce IL-12 canine in tens of milligrams, several factors were evaluated to optimize the production of recombinant canine IL-12 in the baculovirus-insect cell system, as follows, native IL-12 versus the codon optimized IL-12 construct (both as a single-chain protein), the classical and a modified baculovirus vector, multiplicity of infection and time of infection. In addition, it is described a simple and an effective method to semi-quantitatively evaluate expression of secreted recombinant proteins in the baculovírus insect cell system (a dot-blot assay).

## Methods

### Insect cell lines and culture

*Spodoptera frugiperda* and *Trichoplusia ni* insect cell lines, Sf-9 and BTI-TN-5B1-4 (also called High-five), respectively, (Invitrogen, Carlsbad, USA) were used. Sf-9 cells were maintained in TNM-FH medium (Sigma Aldrich, St. Louis, USA) containing 10 % fetal bovine serum (FBS, Invitrogen), 0.1 % Pluronic F-68 solution (Sigma Aldrich), and 10 µg/mL gentamycin sulfate (Sigma Aldrich) (complete TMN-FH medium). High-five cells were cultured in Express-five serum-free medium (SFM) (Invitrogen) supplemented with 16 mM of l-glutamine (Invitrogen), and 10 µg/mL gentamycin (complete Express-five SFM medium). Cell cultures were carried out at 27 °C either as a monolayer or in suspension in shaker flasks, according to the manufacturer’s instructions.

### Interleukin-12 constructs

**Fig. 1:**
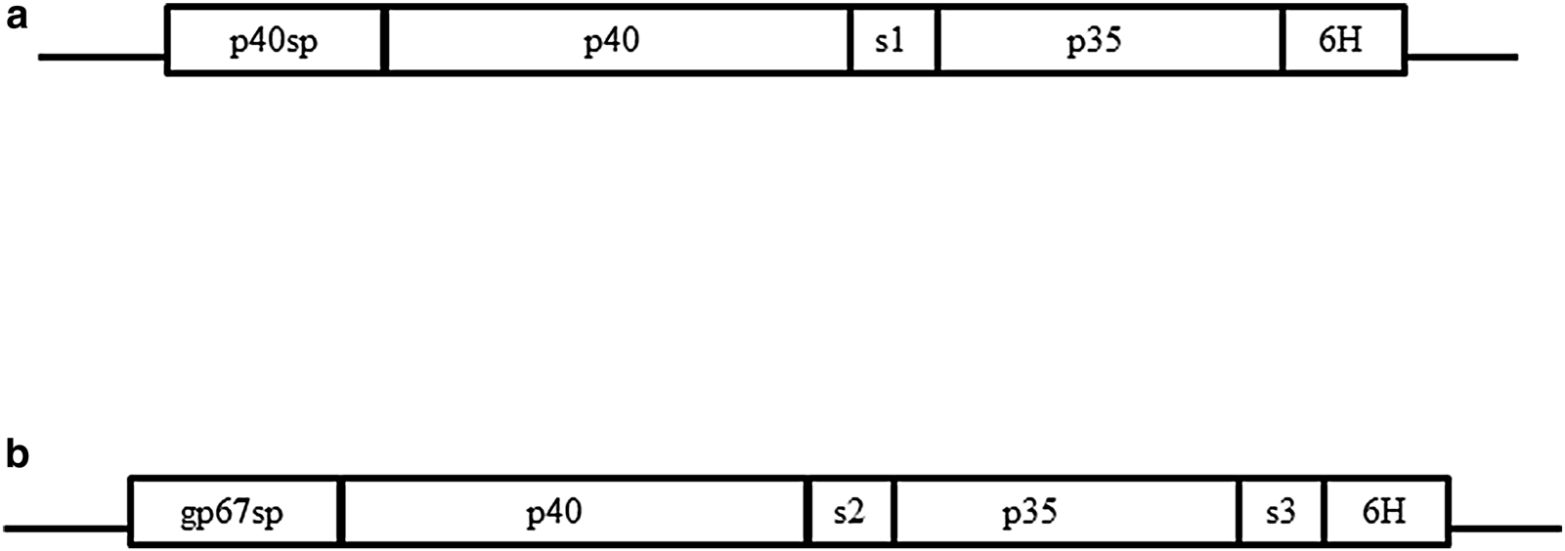
Schematic diagram of DNA constructs encoding canine IL-12 single-chain protein. The construct with canine native DNA is composed in tandem by nucleotide sequences encoding: signal peptide of p40, p40 mature protein, spacer, p35 mature protein and His-tag (**a**); the construct with DNA optimized for *Trichoplusia ni* translation is composed in tandem by nucleotide sequences encoding: GP64 signal peptide of AcMNPV, p40 mature protein, a spacer, p35 mature protein, spacer and His-tag (**b**)

Two DNA constructs encoding canine IL-12 single-chain protein (sccaIL-12 and sccaIL-12opt, and Fig. 1) were cloned into the pFastBac1 plasmid (Invitrogen). The sccaIL-12 construct was originally cloned from native canine mRNA into the pcDNA3.1 plasmid [17] while sccaIL-12opt was synthesized with optimized codons for expression in *T. ni* by Geneart GmBH (Regensburg, Germany). The recombinant proteins encoded by the constructs sccaIL-12 and sccaIL-12opt were named rsccaIL-12S and rsccaIL-12L, corresponding to the length (short or long) of the polypeptide spacer between the p40 and p35 chains, respectively. The successful introduction of inserts into the plasmid pFastBac1, generating the following constructs pFastBac1-sccaIL-12 and pFastBac1-sccaIL12opt, was determined by DNA sequencing.

### Generation of baculovirus encoding canine IL-12

The inserts sccaIL-12 and sccaIL-12opt were transposed from the pFastBac1 plasmid constructs to: (a) Autographa californica multiple nuclear polyhedrosis virus (AcMNPV) bacmid [22] (bacmid named AcBac, Bac-to-Bac system, Invitrogen, cat. 10360-014) and (b) *a chitinase and v*-*cathepsin* knockout bacmid (bacmid called AcBacΔCC, previously described by Kaba and collaborators) [23], using DH10Bac and DH10BacΔCC *Escherichia coli* strains, respectively. Recombinant AcBac-sccaIL-12, AcBac-sccaIL-12opt, AcBacΔCC-sccaIL-12 and AcBacΔCC-sccaIL-12opt baculovirus constructs were obtained by transfecting Sf-9 cells with the corresponding bacmids using a Lipofectamine reagent, following the manufacturer’s instructions (Invitrogen). Baculovirus constructs with the insert from empty pFastBac1 plasmid (Invitrogen) were used as negative controls (AcBac-pFast-cont and AcBacΔCC-pFast-cont). These controls were obtainedthe cell-free SN were stored by transposing the DNA segment between Tn7R and Tn7L from the pFastBac1 plasmid into either the AcBac or AcBacΔCC bacmid and then transfecting Sf-9 cells. Six days after transfection, Sf-9 cell culture suspensions were spun down at 500×*g* for 5 min and baculovírus-containing SN were stored protected from light at 4 °C. To obtain high-titer viral stocks, log phase growing Sf-9 cells (2 × 10^6^/mL) cultured in suspension were infected at a multiplicity of infection (MOI) of 0.1, either for 72 h or until cell viability decreased to approximately 75 %, and then the cell supernatants (SN) were collected as described above. Baculovirus stocks were titrated using the end-point dilution assay [24].

### Optimization of recombinant canine IL-12 expression by insect cells

High-five cells (1 × 10^6^/well) cultured as monolayers in serum free medium in 6-well titration plates were infected with the recombinant baculovirus constructs (AcBac-sccaIL-12, AcBac-sccaIL-12opt, AcBacΔCC-sccaIL-12, or AcBacΔCC-sccaIL-12opt) at MOI of 2, 5 or 10. To assess secreted recombinant protein, the cell culture SN were collected at 24, 48 and 72 h post infection, floating cells were spun down at 500×*g* for 5 min, and the cell-free SN were stored at −20 °C until use. Recombinant canine IL-12 in the cell culture SN was evaluated by dot-blot assay, using mouse anti-histidine C-terminal antibodies (mouse anti-His-C Ab, Invitrogen) as described below.

### Dot-blot and Western blot analysis

For dot-blot assays, nitrocellulose membranes placed in a blotter device (BioRad, Hercules, United States) were directly coated with proteins from cell culture SN at 150 µL/well. The membranes were blocked with tris-buffered saline, pH 7.6, containing 5 % powdered non-fat milk and 0.05 % Tween 20 and developed with mouse anti-His-C Ab (Invitrogen) diluted 1:5.000, goat anti-mouse IgG alkaline phosphatase-conjugate diluted 1:500 (Sigma-Aldrich), and alkaline phosphatase substrate (5-bromo-4-chloro-3-indolyl-phosphate and nitro blue tetrazolium, Sigma Aldrich). For Western blot analysis, samples of SN or cell lysate from similar volumes of cultures of High-five cells infected with AcBac-pFast-cont, AcBacΔCC-pFast-cont, AcBac-sccaIL-12, AcBacΔCC-sccaIL-12, AcBac-sccaIL-12opt or AcBacΔCC-sccaIL-12opt baculovirus contructs at a MOI 5 for 48 h were fractioned by polyacrylamide gel electrophoresis with dodecyl sodium sulfate (SDS–PAGE) and then transferred to nitrocellulose membranes (BioRad). Detection of his-tagged protein was performed as described in the dot-blot assay.

### Production of recombinant canine IL-12 by High-five cells

Production of recombinant canine IL-12 in High-five cells was carried out in 50 mL suspension cultures using complete Express-five-SFM. For this, exponentially growing cells were adjusted to 2 × 10^6^/mL and then infected with AcBac∆CC-caIL-12opt at MOI 5. After 48 h of infection, cell suspension was centrifuged at 3.000×*g* for 15 min, cell-free SN was spun down at 30.000×*g* for 1 h to remove the virus particles and, the resulting SN was stored at −70 °C until use. The virus-free SN was dialyzed twice against 0.15 M phosphate-buffered saline, pH 7.8 (PBS) and once against binding buffer (20 mM Na_2_HPO_4_, 500 mM NaCl, 20 mM imidazole, pH 7.8), and then applied onto Histrap HP columns (GE Healthcare, Uppsala, Sweden) attached to an AKTA Purifier chromatography system (GE Healthcare). The column was washed with binding buffer and eluted using an imidazole linear gradient from 20 to 500 mM in 20 mM Na_2_HPO_4_, 500 mM NaCl, pH 7.8, buffer, following the manufacturer’s recommendations. The purified protein (rsccaIL-12L) was buffer exchanged to PBS using a Hitrap desalting column (GE Healthcare) and stored at −70 °C until use. Protein samples were assessed by SDS–PAGE using 12 % polyacrylamide gels and previously described Western blotting assay. A molecular weight calibration curve was prepared and molecular weight sizes of protein bands were estimated using GraphPad Prism v.5.0 software (GraphPad Prism Inc., San Diego, CA). In addition, the protein and endotoxin concentrations were determined using Micro BCA protein assay kit (Thermo Scientific, Rockford, USA, cat. 23235) and *Limulus* amebocyte lysate method (Lonza, Walkersville, USA, cat. 50-647U), respectively.

### Circular dichroism measurements

Circular dichroism spectra of rsccaIL-12L were obtained at 0.7 mg/mL protein concentration. Far-UV spectra were measured from 190 to 260 nm, averaged over three scans at a speed of 50 nm/min, and collected in 1 nm steps. The buffer baselines were subtracted from their respective sample spectra. These measurements were carried using a Jasco J-815 spectropolarimeter (Jasco Corp., Tokyo, Japan) with a 0.2 cm path-length quartz cuvette.

### Fluorescence spectroscopy measurements

Tryptophan fluorescence emission spectra were obtained and recorded by setting the excitation wavelength at 280 nm and the emission spectrum was recorded from 295 to 415 nm using a Jasco FP-6500 spectrofluorimeter (Jasco Corp.).

### Assessment of interferon gamma (IFN-γ) production by peripheral blood mononuclear cells (PBMC) stimulated with purified recombinant canine IL-12

Peripheral blood samples collected from six healthy adult mongrel dogs (three males and three females) were used. These animals were donated while still puppies to Gonçalo Moniz Research Center kennel by their owners. Thereafter the animals were housed at Gonçalo Moniz Research Center kennel. The experiments were performed in accordance with the guidelines of the Oswaldo Cruz Foundation for laboratory animal use and approved by Gonçalo Moniz Research Center Ethical Committee (Licence Protocol 021/2011‐CPqGM). The concentration of IFN-γ was measured in peripheral blood mononuclear cells (PBMC) culture SN by capture ELISA using a protocol previously described by Pereira and collaborators [27]. Briefly, PBMC purified in a Ficoll-Paque PLUS (GE Healthcare) gradient were suspended in RPMI-1640 medium (Sigma Aldrich) supplemented with 10 % FBS, 10 mM HEPES (Invitrogen), pH 7.0, 2 mM l-glutamine (Sigma Aldrich) and 50 µg/mL gentamycin (complete RPMI medium). One hundred microliters of PBMC suspension at 2 × 10^5^/mL were placed per well in a 96-well flat-bottom microtritation plate. One hundred µL/well of the following solutions were added to four wells: (a) complete RPMI, (b) SN of culture of COS-7 cells transfected with a plasmid encoding canine IL-2 (pcDNA3.1-caIL-2) [27] diluted 1:10 (COS-7 cells caIL-2 SN), (c) rsccaIL-12L at 40 ng/mL, or (d) rsccaIL-12L at 40 ng/mL in combination with COS-7 cells caIL-2 SN diluted 1:10. Then PBMC were cultured in a humidified atmosphere with 5 % CO_2_, at 37 °C, for 48 h. After that, supernatants of each quadruplicate were pooled, spun down at 300×*g* for 5 min to remove the cells and stored at −20 °C until use. The concentration of IFN-γ in the SN was determined by capture ELISA using specific antibodies produced by R&D Systems (Minneapolis, USA), according to the manufacturer’s instructions. A calibration curve was prepared with recombinant IFN-γ (R&D Systems) following the manufacturer’s instructions and GraphPad Prism v.5.0 software.

### Statistical analysis

The values for IFN-γ concentration were compared by Repeated Measures ANOVA followed by Dunnett’s post-test. Values of p < 0.05 were considered significant. The statistical analysis was performed and graphs made using GraphPad Prism v.5.0 software.

## Results

### Expression of recombinant canine IL-12

To confirm transfer of the inserts (sccaIL-12, sccaIL-12opt, and the segment between Tn7R and Tn7L from empty vector) from pFastBac1 plasmid constructs or pFastBac1 empty plasmid to either AcBac or AcBacΔCC bacmid, PCR was carried with primers flanking the bacmid transposition sites. Amplicons produced by PCR showed the expected size (data not shown), indicating success in obtaining the recombinant (AcBac-sccaIL-12, AcBacΔCC-sccaIL-12, AcBac-sccaIL-12opt, AcBacΔCC-sccaIL-12opt) and negative control (AcBac-pFast-cont, and AcBacΔCC-pFast-cont) bacmids.

**Fig. 2:**
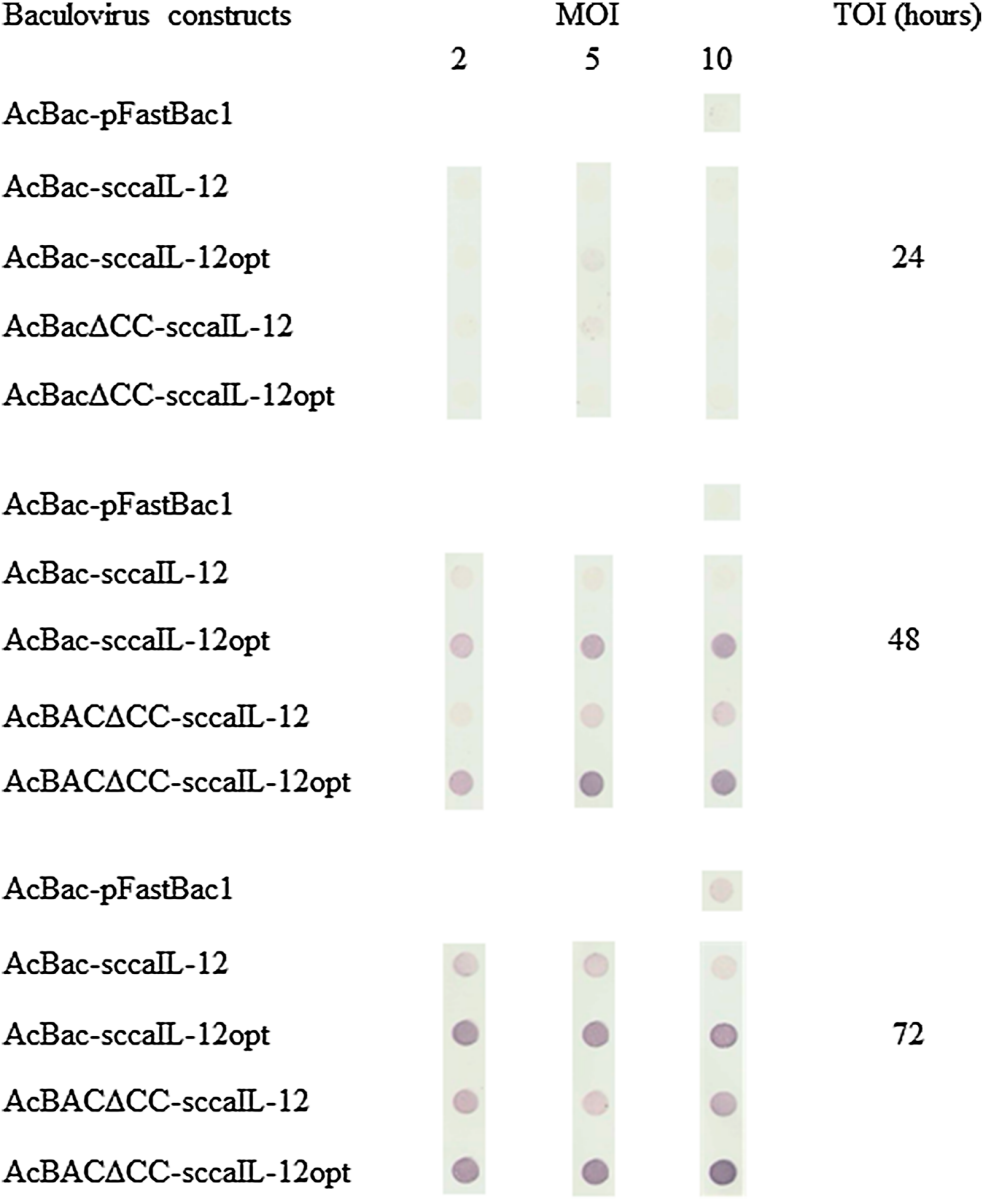
Optimization of canine IL-12 single-chain protein expression in the baculovírus-insect cell system. High-Five cells were infected with distinctive baculovírus constructs using different multiplicity of infection (MOI) and time of infection (TOI). Dot-blot assay was carried out using 150 μL of infected cell culture supernatant proteins impregnated on nitrocellulose membrane and anti-His C-Term antibodies followed by anti-mouse immunoglobulin-alkaline phosphatase conjugate and substrate

To determine the best conditions for recombinant canine IL-12 expression in insect cells, High-five cells were infected with each of the four recombinant baculovirus constructs at MOI 2, 5 and 10, or one of the negative controls at MOI 10, and culture SN were assessed by dot-blot analysis at 24, 48 and 72 h post-infection (pi), using an antibody specific for C-terminal his-tags. Taking into account the background signal observed when SN of High-five cells cultured with AcBac-pFast-cont baculovirus was utilized at MOI 10 (Fig. 2), cells infected with recombinant baculovirus constructs started producing recombinant canine IL-12 after 48 h pi. Interestingly, the largest amounts of cytokine were secreted when the High-five cells were infected with the AcBac-sccaIL-12opt and AcBacΔCC-sccaIL-12opt baculovirus constructs, whose inserts had codons optimized for expression in *Trichoplusia ni*, at MOI 2 72 h pi, MOI 5 48 or 72 h pi, and MOI 10 48 or 72 h pi (Fig. 2). High-five cells also synthesized the recombinant protein when infected with AcBac-sccaIL-12 and AcBacΔCC-sccaIL-12 constructs, but at a lower level comparing to the cells exposed to baculovirus constructs carrying the codon-optimized insert (AcBac-sccaIL-12opt and AcBacΔCC-sccaIL-12opt) (Fig. 2). High-five cells infected with AcBac-sccaIL-12, AcBacΔCC-sccaIL-12, AcBac-sccaIL-12opt, and AcBacΔCC-sccaIL-12opt baculovirus constructs at MOI 5 for 48 h were selected as the conditions for further studies.

### Baculovirus constructs containing sccaIL-12opt promote efficient production of recombinant canine IL-12 by High-five cells

To assess the efficiency of secretion of recombinant canine IL-12, cell culture SN and lysates of High-five cells infected with either AcBac-sccaIL-12, AcBacΔCC-sccaIL-12, AcBac-sccaIL-12opt, AcBacΔCC-sccaIL-12opt, AcBac-pFast-cont or AcBacΔCC-pFast-cont baculovirus constructs at MOI 5 for 48 h were evaluated by Western blot with anti-His C-term antibodies.

**Fig. 3:**
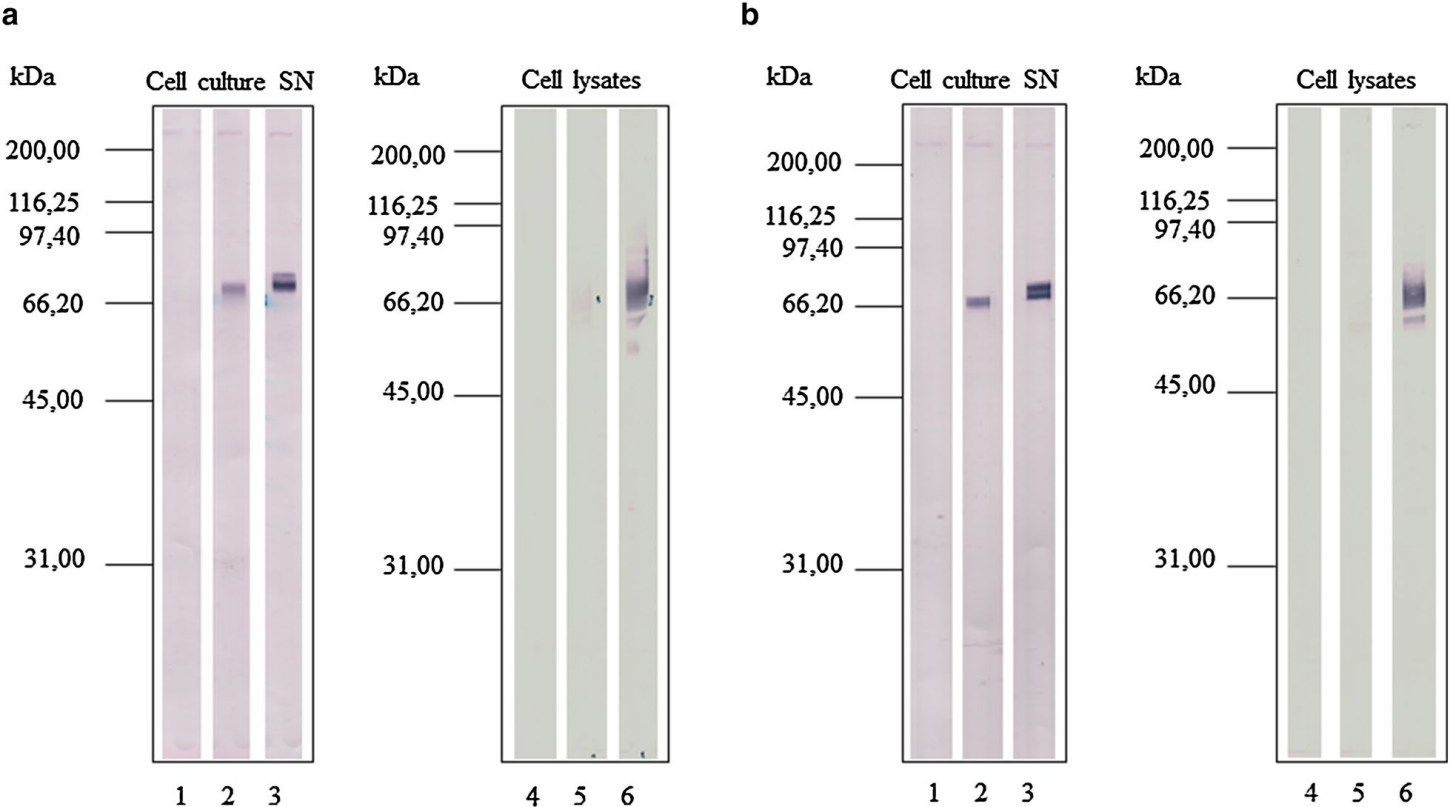
Detection of canine IL-12 single-chain protein produced by infected High-five cells by Western blot. High five cells were infected with the following baculovirus constructs AcBac-pFast-cont (*lanes 1, 4*), AcBac-sccaIL-12 (*lanes 2, 5*), AcBac-sccaIL-12opt (*lanes 3, 6*) (**a**) or AcBacΔCC-pFast-cont (lanes 1, 4), AcBacΔCC-sccaIL-12 (*lanes 2, 5*), AcBacΔCC-sccaIL-12opt (*lanes 3, 6*) (**b**), at multiplicity of infection 5 for 48 h. Proteins from supernatants and lysates from the same volume of cell culture were fractioned into SDS–PAGE and transferred to nitrocellulose membrane. Membrane strips were incubated with mouse anti-his C-Term antibodies, followed by anti-mouse immunoglobulin-alkaline phosphatase conjugate and substrate

In Western blot analysis, culture SN of cells infected with AcBac-sccaIL-12 or AcBacΔCC-sccaIL-12 baculovirus construct showed a protein band with a molecular mass of 67 kDa (Fig. 3a, b, lanes 2), which was absent in the negative control SN (Fig. 3a, b, lanes 1). In addition, the culture SN of cells infected with either the AcBac-sccaIL-12opt or the AcBacΔCC-sccaIL-12opt baculovirus construct showed two strong bands with molecular masses of 67 and 70 kDa, being these bands strongest when AcBacΔCC-sccaIL-12opt construct was used (Fig. 3a, b, lanes 3). Therefore, the amount of IL-12 detected in the SN of cells infected with either baculovirus construct carrying the codon optimized insert (AcBac-sccaIL-12opt and AcBacΔCC-sccaIL-12opt) was higher than in the SN of cells cultured with baculovirus constructs carrying native canine IL-12 cDNA (AcBac-sccaIL-12 and AcBacΔCC-sccaIL-12).

Analyzed by Western blot, lysates of cells infected with AcBac-sccaIL-12 or AcBacΔCC-sccaIL-12 showed a weak broad band of average molecular mass of 67 kDa and a weak band of 61 kDa, respectively,which were not seen -in negative control lysates. Lysates of cells infected with AcBac-sccaIL-12opt and AcBacΔCC-sccaIL-12opt baculovirus constructs displayed a strong broad band with an average molecular mass of 67 kDa and a weak band of 61 kDa (Fig. 3), which were not present in the negative control lysates. In addition, lysate of High-five cells infected with AcBac-sccaIL-12opt presented a weak band of 54 kDa (Fig. 3), absent in the corresponding negative control lysate.

Western blot results suggest that the AcBacΔCC-sccaIL-12opt recombinant baculovirus construct induced the highest amount of secreted recombinant canine IL-12 (rsccaIL-12L) and more than half of the total recombinant protein produced was retained within the cells.

**Fig. 4:**
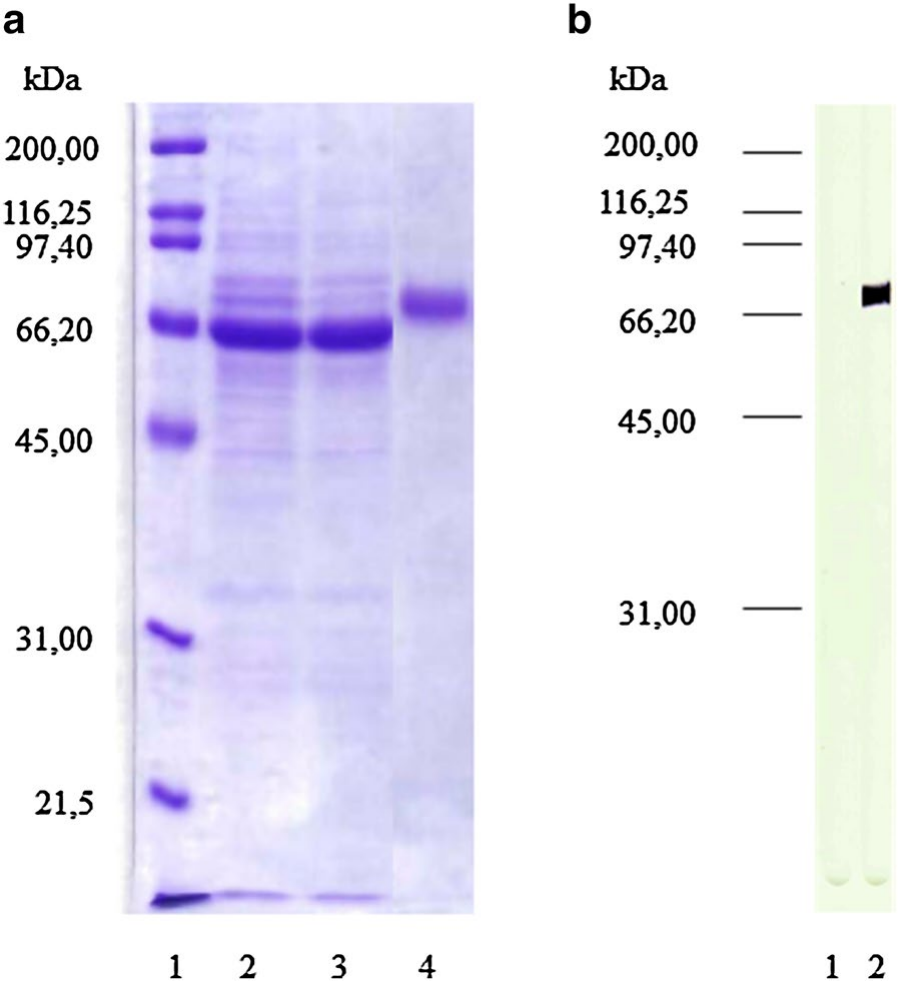
Analysis of purified rsccaIL-12L produced in the baculovirus-insect cell system by SDS–PAGE and Western blot. Recombinant sccaIL-12L was produced by High-five cells infected with the AcBacΔCC-sccaIL-12opt baculovirus construct at MOI 5 for 48 h. Clarified and buffer-exchanged cell culture supernatant was applied to Ni-Sepharose affinity chromatography column and the recombinant protein was eluted using an imidazole linear gradient. The chromatographic fractions were analyzed by SDS–PAGE and Western blot. Polyacrylamide gel showing: molecular weight markers (*lane 1*), clarified buffer-exchanged cell culture supernatant applied to the chromatographic column (10 μL, *lane 2*), flow through fraction (10 μL, *lane 3*), and sample of a chromatographic fraction containing rsccaIL-12 (10 μL, *lane 4*) (**a**). Nitrocellulose membrane showing purified sccaIL-12 incubated with normal mouse serum diluted 1:500 (*lane 1*) or mouse anti-his C-Term antibodies (*lane 2*), followed by anti-mouse immunoglobulin-alkaline phosphatase conjugate and substrate (**b**)

To determine the yield and biological activity, rsccaIL-12L was purified from the SN of High-Five cells infected with AcBacΔCC-sccaIL-12opt baculovirus construct at MOI 5 for 48 h. After purification from the cell SN by affinity chromatography, rsccaIL-12L displayed a single band with molecular weight size of 68.5 kDa in Western blot and SDS–PAGE analysis (Fig. 4). Considering the amount of the recombinant protein purified from 50 mL of cell culture SN, it was estimated a yield of 6 mg of rsccaIL-12L per liter of culture. Purified rsccaIL-12L had 0.1 EU of endotoxin per mg of protein.

**Fig. 5:**
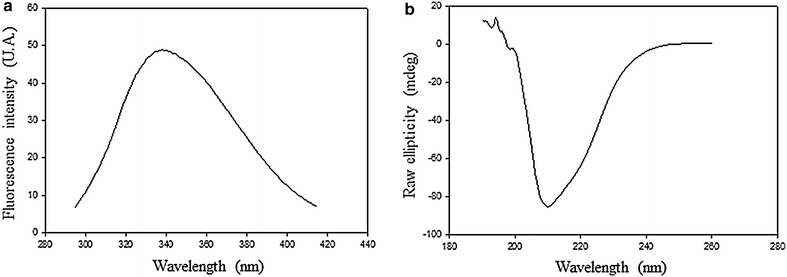
Fluorescence and circular dichroism of rsccaIL-12L. Intrinsic fluorescence spectra of rsccaIL-12L obtained by excitation at 280 nm, and emission from 295 to 415 nm (**a**). Circular dichroism spectra of rsccaIL-12L, at 190–260 nm, resulting from three consecutive measurements at the speed of 50 nm/min (**b**)

The fluorescence spectrum of rsccaIL-12L showed maximum peak in 338 nm (Fig. 5a), suggesting that the recombinant protein was properly folded. In addition, circular dichroism analysis revealed a spectrum with a maximum negative ellipticity at 210 nm, indicating that the target protein has a predominantly β-sheet structure (Fig. 5b).

### Recombinant canine sccaIL-12L produced by High-five cells induces IFN-γ in homologous PBMC

**Fig. 6:**
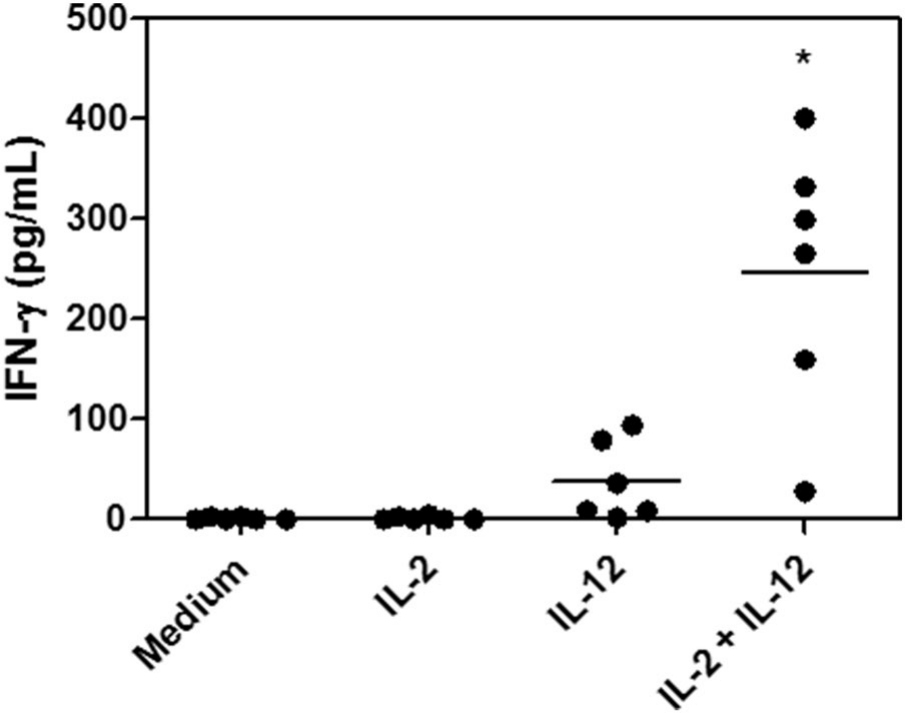
Assessment of IFN-γ production by peripheral blood mononuclear cells (PBMC) stimulated with rsccaIL-12L. PBMC from six adult mongrel dogs were cultured for 48 h with: complete RPMI 1640 medium alone (medium), COS-7 cells supernatant containing canine IL-2 at 20 % (IL-2), purified rsccaIL-12L at 20 ng/mL (IL-12); or purified sccaIL-12L at 20 ng/mL combined with COS-7 cell SN-caIL-2 (IL-2 + IL-12). IFN-γ was assessed in the PBMC supernatants by capture ELISA. Data represent the average of duplicates and means obtained from six animals. Repeated measures ANOVA: p < 0.0001, Dunnett´s test *p < 0.05

The biological activity of rsccaIL-12 was evaluated by testing its ability to induce IFN–γ production in PBMC from six healthy adult dogs. Canine PBMC were cultured for 48 h with:(a) complete RPMI, (b) COS-7 cells caIL-2 SN, (c) rsccaIL-12L, or (d) rsccaIL-12L in combination with COS-7 cells caIL-2 SN. IFN-γ was measured in culture SN by capture ELISA. Canine PBMC have not produced IFN-γ when cultured in complete RPMI (mean ± SD, 1 ± 1 pg/mL) or in the presence of SN COS-7 containing IL-2 (1 ± 2 pg/mL). PBMC of 3 out of 6 dogs produced IFN-γ when cultured with rsccaIL-12L at 20 ng/mL, however, the mean concentration (mean ± SD, 38 ± 40 pg/mL) observed in the SN of stimulated cells was not significantly different from the SN of cells cultured in medium alone (Fig. 6). Nevertheless, when PBMC were stimulated with both rsccaIL-12L at 20 ng/ml and SN COS-7 containing IL-2, cells of 6 out of 6 dogs produced significantly higher amounts of IFN-γ (247 ± 134 pg/mL) than cells cultured with complete RPMI (Fig. 6), indicating that purified rsccaIL-12L was biologically active.

## Discussion

IL-12 has the potential to promote, alone or in combination with other immunomodulatory agents, cell-mediated immune responses [9, 25–27], which are useful in the control of infections caused by intracellular pathogens, of allergic diseases mediated by antibody [28], and of neoplasms [26]. Aiming at optimizing conditions to produce tens of milligrams of canine IL-12 for future pre-clinical trials in dogs, several parameters were evaluated in the present work, including different baculovirus constructs, and time of infection (TOI 24, 48 and 72 h) and multiplicity of infection (MOI 2, 5 and 10) of High-five insect cells.

In order to obtain a good yield of secreted mammalian recombinant protein in the baculovirus-insect cell system, DNA constructs have been used with: (a) elements potentially capable of promoting translation or secretion or (b) baculovirus vector framework knocked out of genes encoding proteins targeted to endoplasmic reticulum. For example, to enhance translation, constructs have been made with: (a) AT rich nucleotide sequence before the initiation codon and an adenine at position +4, that would promote recognition of mRNA by ribosomes in insect cells, instead of Kozak sequence [29–31], (b) codons specifically optimized for the species of the used host [32]. To improve secretion, constructs have been prepared with: (a) signal peptide encoding sequences from proteins efficiently targeted to endoplasmic reticulum of insect cells, like AcMNPV GP64 protein or honeybee venom melittin, instead of native signal peptide [31, 33], or (b) a baculovirus vector from which several genes were deleted, for example, chitinase, cathepsin, p10, p26 and p74 [23, 34–36], which encode proteolytic enzymes or proteins capable of competing for secretory machinery resources of the host cell.

In the current study, a recombinant baculovirus construct (AcBac-sccaIL-12) was used with the classic baculovirus vector framework (AcBac) and canine native DNA encoding IL-12 (sccaIL-12). In addition, recombinant baculovirus constructs were used with: (a) AcBac knockout of chitinase and cathepsin genes (AcBacΔCC) and sccaIL-12 insert (AcBacΔCC-sccaIL-12), (b) AcBac and an insert comprised of DNA encoding AcMNPV GP64 signal peptide and canine IL-12 mature polypeptides, with optimized codons for translation in *T. ni* (sccaIL-12opt) (AcBac-sccaIL-12opt) and, (c) AcBacΔCC and sccaIL-12opt (AcBacΔCC-sccaIL-12opt). Furthermore, constructs were assessed by their ability to promote IL-12 secretion in High-Five cells to determine if any of the changes made in basic construct design was advantageous.

The constructs were evaluated initially by assessing culture SN of High Five cells infected with MOI 2, 5 or 10 and TOI 24, 48 or 72 h, using the dot-blot assay. All baculovirus constructs were able to induce the secretion of both rsccaIL-12S and rsccaIL-12L recombinant proteins. However, AcBac-sccaIL-12opt and AcBacΔCC-sccaIL-12opt constructs induced the largest quantities of rsccaIL-12L- the latter being slightly more efficient than the former. These results suggest that the use of: (a) AcMNPV GP64 signal peptide, in contrast to the native signal peptide, and/or (b) codons optimized for *T. ni* (in contrast to the canine native codons), and (c) a baculovirus vector devoid of cathepsin and chitinase genes (in contrast to the classical baculovirus vector), contributed to increased production of secreted recombinant protein. Factors described in “a” and “b” had a greater impact than the one mentioned in “c”. Interestingly, in one experiment in which production of secreted recombinant canine IL-12 was assessed by dot-blot of the supernatant of Sf-9 cells infected with MOI 2, 5 and 10 and TOI of 24, 48 and 72 h, the four baculovirus constructs were equally efficient (data not shown), suggesting that replacement of the native signal peptide for that of AcMNPV GP64, optimizing codons for *T. ni* and the use of a baculovirus vector devoid of cathepsin and chitinase genes have no impact on expression in Sf-9 cells. These results are in agreement with reports by several authors which show that certain changes in the insert and in the genome of the baculovirus vector itself can promote an increased yield of recombinant proteins and that the same baculovirus construct may promote different yields in different host cells, e.g., Sf9 and High-Five cells [37–39]. Therefore, the baculovirus-insect cell system is not fully predictable [33].

Various methods have been used for detection of secreted recombinant proteins in the process of optimizing production conditions in baculovirus-insect cell system, including ELISA [37, 39, 40], enzyme assay [41, 42] and Western blot analysis [23, 43, 44]—the latter being most commonly used. Western blot analysis is a labor-intensive method that involves several steps, including separation in polyacrylamide gel and transfer to nitrocellulose membrane, before protein detection. In the current study, a simpler method (dot-bot) was successfully used to optimize production conditions of a recombinant protein secreted in the culture SN of cells grown in serum-free medium. The best conditions for production of the recombinant protein were MOI 5 and TOI 48 h. These conditions were chosen taking into account the difference between the signal to noise ratio observed in the dot-blot assay. In our laboratory, dot-blot analysis was also successfully used to optimize production of three other secreted recombinant proteins in the baculovirus-insect cell system (unpublished data).

To determine the secretion efficiency and integrity of the recombinant canine IL-12 produced by High-five cells infected with each of the four recombinant baculovirus constructs at the best conditions, Western blot assays were carried out using samples of the culture SN and cell lysates. Taking into account the presence and intensity of bands with molecular masses between 67 and 70 kDa detected by anti-His tagged antibody, the total production (sum of the recombinant protein in culture SN and cell lysate) and secretion of the recombinant canine IL-12, it can be concluded that the recombinant protein was obtained in progressively larger amounts by the constructs AcBac-sccaIL-12, AcBacΔCC-sccaIL-12, AcBac-sccaIL-12opt and AcBacΔCC-sccaIL-12opt. Data concerning detection of the recombinant protein in culture SN corroborate the findings of the dot-blot assay, confirming that the use of the GP64 signal peptide and the codons optimized for *T. ni* had the greatest impact and that the baculovirus vector framework devoid of cathepsin and chitinase genes impacted only modestly on the production of the secreted IL-12.

For unknown reasons, when the constructs AcBac-sccaIL-12opt and AcBacΔCC-sccaIL-12opt were used, apparently, most of the translated protein was retained inside the host cells (Fig. 3). A few studies have also shown that sometimes, especially while using High-Five cells, baculovirus constructs have the potential to induce secretion of tens to hundreds of milligrams of recombinant proteins per liter of cell culture [39, 45]. Since the yield of canine IL-12 observed in this study was lower than 10 mg/L of culture, it is improbable that the secretory machinery was overwhelmed to explain the retention of most of the recombinant cytokine within the host cells. Many authors have also observed retention of secretable recombinant protein inside the cells when using the baculovirus-insect cell system [23, 39, 46, 47]. The presence of a small number of bands detected in the cell lysate by Western blot, being the majority of bands detected with molecular masses above the predicted mass for the mature protein (rsccaIL-12S, 58.3 kDa and rsccaIL-12L, 61.3 kDa), suggests that there was little, if any, proteolysis of the recombinant protein during the production process. The two bands (67 and 70 kDa) observed in culture SN of cells infected with AcBac-sccaIL-12opt and AcBacΔCC-sccaIL-12opt baculovirus constructs probably represent glycosylated isoforms of rsccaIL-12L [23]. Assessment of glycosylation of rsccIL-12L is underway.

In view of the results obtained from dot blot and Western blot assays, the baculovirus construct AcBacΔCC-sccaIL-12opt was used to infect High-Five cells at MOI 5 and TOI 48 in order to produce recombinant canine IL-12 for purification and biological evaluation.

To determine the quality, samples of the purified protein were analyzed by SDS–PAGE, Western blot, fluorescence emission spectrum, circular dichroism, and for endotoxin contamination. Evaluation carried out by SDS–PAGE with gel stained with Coommassie blue and Western blotting showed a single band with an average molecular mass of 68.5 kDa, instead of the expected two bands of 67 and 70 kDa, previously detected during analysis of culture SN. This was probably related to the greater amount of protein used in the previous experiments making the bands appear fused. Assessment by the fluorescence emission spectrum and circular dichroism revealed data compatible with a folded protein predominantly composed of beta sheets. These findings are consistent with data previously reported for human IL-12 [48, 49]. Endotoxin was detected only in very low concentrations. Finally, determination of protein concentration carried out in samples of two independent experiments allowed the estimation of a yield of 6–9 mg of sccaIL-12L per liter of insect cell culture. Therefore, taking into account protein purity, integrity, folding, endotoxin contamination, and yield, the recombinant protein was successfully purified.

The functional activity of the purified protein (rsccaIL-12L) was determined by its ability to induce the synthesis of IFN-γ in canine PBMC. Previous studies reported the requirement of dual stimulation, using both IL-12 and IL-2, for the induction of IFN-γ secretion by canine PBMC [27]. In the current study, PBMC from healthy dogs were cultured with rsccaIL-12L alone or in combination with COS-7 SN containing IL-2, the latter at a concentration that was unable to induce IFN–γ synthesis by itself. Stimulation with rsccaIL-12L alone induced IFN-γ production above background levels in 3 out of 6 dogs, although this was not statistically significant. On the other hand, stimulation with rsccaIL-12L in combination with COS-7 SN containing IL-2 resulted in synthesis of IFN-γ by PBMC from all six dogs tested, and compared with PBMC cultured with COS-7 SN containing IL-2 alone or medium alone, this was statistically significant, thus, indicating that the recombinant protein produced was biologically active. Previously, Poot et al. [21] have produced canine IL-12 in the baculovirus-insect cell system and shown it is able to induce proliferation of equine lymphoblasts. Unfortunately, these authors have not assessed the ability of canine IL-12 to stimulate the IFN-γ synthesis in canine PBMC that would allow some comparison with the current work.

In conclusion, this is the first description of a method for production of bioactive canine IL-12 in suitable amounts for pre-clinical trials. In addition, the combination of several factors (use of AcMNPV GP64 signal sequence, codons optimized to insect cells, and baculovirus knockout for chitinase and cathepsin) described in the current manuscript has a wide application in the field of recombinant protein production, useful for future studies on prophylactic and therapeutic interventions in cancer, inflammatory and infectious diseases in dogs.

## Abbreviations

AcBac-pFast-cont: control classical baculovirus with no canine IL-12 insert
AcBacΔCC-pFast-cont: control *chitinase A and v*-*cathepsin* knockout baculovirus with no canine IL-12 insert
AcBac-sccaIL-12: classical baculovirus containing a native insert of canine IL-12
AcBac-sccaIL-12opt: classical baculovirus containing optimized insert of canine IL-12
AcBacΔCC-sccaIL-12: *chitinase A and v*-*cathepsin* knockout baculovirus containing a native insert of canine IL-12
AcBacΔCC-sccaIL-12opt: *chitinase A and v*-*cathepsin* knockout baculovirus containing an optimized insert of canine IL-12
His: histidine
pFastBac1: empty vector
pFastBac1-sccaIL-12: vector containing a native insert of canine IL-12
pFastBac1-sccaIL12opt: vector containing optimized insert of canine IL-12
sccaIL-12: native insert of canine IL-12 as single-chain protein
sccaIL-12opt: optimized insert of canine IL-12 as single-chain protein
SN: supernatants
